# Editorial: Precise chemical application technology for horticultural crops

**DOI:** 10.3389/fpls.2024.1413941

**Published:** 2024-05-28

**Authors:** Xue Li, Xiaolan Lv, Andreas Herbst, Jianli Song, Tao Xu, Yannan Qi

**Affiliations:** ^1^ Institute of Agricultural Facilities and Equipment, Jiangsu Academy of Agricultural Sciences, Nanjing, Jiangsu, China; ^2^ Key Laboratory of Horticultural Equipment, Ministry of Agriculture and Rural Affairs, Nanjing, China; ^3^ Application Techniques in Plant Protection, Julius Kühn Institute, Braunschweig, Germany; ^4^ College of Science, China Agricultural University, Beijing, China

**Keywords:** chemical application, horticultural crops, plant protection, precision agriculture, spraying

The field of plant chemical protection utilizes professional spraying machines to transport chemicals, such as pesticides and herbicides, to targeted sites for effective control of pests, diseases, and weeds in horticultural crop production (Wang et al., 2022). These practices play a crucial role in ensuring the provision of safe and abundant food, fruits, and vegetables for human consumption. However, conventional methods of applying plant chemicals suffer from issues such as excessive pesticide usage, low utilization rates, and high labor intensity due to outdated sprayers and single atomization technology (Abbas et al., 2020). To further advance modern agriculture, it is necessary to focus on efficient, precise, and intelligent application technologies and equipment for plant chemical protection. By incorporating smart chemical application technology with efficient equipment, significant reductions in pesticide usage can be achieved while mitigating environmental harm caused by chemicals and alleviating labor intensity (Talaviya et al., 2020). This approach facilitates sustainable development in disease and pest control of horticultural crops while ensuring food safety as well as maintaining crop yield and quality.

The published articles cover three main areas: target detection, variable-rate automated spraying systems, and autonomous trajectory planning and navigation. The primary goal of this Research Topic is to comprehensively study novel technologies for applying chemicals in horticultural crop cultivation. This focused examination will facilitate the exploration of innovative theories, advancements in technological solutions, and optimization of chemical applications for effective pest control, disease prevention, and weed management in horticultural crops.

## Target detection

The precision targeting technology is essential for implementing precise variable-rate spraying. Before applying variable-rate spraying, agricultural machinery needs to accurately detect and acquire the target or application area, and then execute automatic spraying based on the severity of pests and diseases (Li et al., 2017). The essence of precision targeting technology lies in target detection. Currently, various methods such as infrared, ultrasonic, lidar, machine vision, spectral imaging, and multi-sensor fusion are employed for detecting targets (Partel et al., 2021).

Qiu et al. proposed a citrus disease classification model that incorporates data diversity, knowledge assistance, and modal fusion. The study utilized leaves from healthy plants as well as plants infected with 10 prevalent diseases to construct three datasets with white, natural, and mixed backgrounds. This approach aimed to enhance the adaptability of the model in real-world scenarios. Visual features of the leaves were extracted using convolutional networks of varying depths (VGG16, ResNet50, MobileNetV2, and ShuffleNetV2). Additionally, TextCNN and fastText were employed to extract textual features and semantic relationships. By integrating image and text information synergistically, a joint learning model for citrus disease features was developed. The results demonstrated that ShuffleNetV2 + TextCNN achieved an impressive classification accuracy of 98.33% on the mixed dataset – an improvement of 9.78% over single-image models and 21.11% over single-text models respectively. Furthermore, this model exhibited faster convergence rate, superior classification balance, and enhanced generalization capability compared to other methods available in literature; thus, providing a more reliable basis for decision-making regarding precise application of biological and chemical control strategies in citrus production.The dataset created by Lyu et al. consists of images capturing citrus psyllids in their natural habitats, and they proposed a lightweight detection model based on spatial channel interaction. Experimental results demonstrated that the YOLO-SCL model exhibits excellent accuracy in detecting citrus psyllids. Compared to the conventional YOLOv5s model, the YOLO-SCL model achieved a mAP@0.5 of 97.07% for citrus psyllids, surpassing the performance of the conventional YOLOv5s model by 1.18%. Furthermore, the number of parameters and computational requirements of the YOLO-SCL model are reduced by 14.25% and 2.52%, respectively, compared to those of the conventional YOLOv5s model. Additionally, through optimization using the black widow algorithm for hyperparameters, the mAP@0.5 score for citrus psyllid detection with the YOLO-SCL model improved to 97.18%, making it more suitable for detecting these pests in natural environments.

## Variable-rate automated spraying systems

The variable-rate automated spraying technology for pesticides plays a vital role in achieving accurate pesticide application by gathering data on factors including pest severity, crop canopy morphology and density (Tumbo et al, 2007). This information is then integrated with operational parameters such as position, speed, and spray pressure to precisely target crops.

Xue et al. designed a variable spraying control system. The system employed a Kinect sensor to real-time detect the canopy volume of citrus trees and adjusted the duty cycle of solenoid valves by pulse width modulation to control the pesticide application. The experimental results indicated that the variable spraying control system demonstrated good consistency between the theoretical spray volume and the actual spray volume. Compared to constant-rate spraying, the droplets under the variable-rate mode based on canopy volume exhibited higher deposition density achieving the goal of reducing pesticide use with a maximum pesticide saving rate of 57.14%.

## Autonomous trajectory planning and navigation

The autonomous navigation of agricultural machinery relies on machine vision, lidar, and satellite positioning techniques. Machine vision extracts reference data from ground oblique views and aerial global views. Lidar technology establishes the operational environment through SLAM methods. Satellite positioning transmits geographic information to enable navigation via wireless data transmission.

Traditional navigation methods based on GNSS and 2D LlDAR is unreliable in complex scenarios with little sensory information due to tree canopy occlusion. Xia et al. proposes a 3D LiDAR-based navigation method for trellis orchards. Using 3D LiDAR with a 3D SLAM algorithm, orchard point cloud information was collected and filtered using the PCL (point cloud library) to extract trellis point clouds as matching targets. In terms of positioning, the real-time position was determined through a reliable method of fusing multiple sensors for positioning, which involves transforming the RTK information into the initial position and doing a normal distribution transformation between the current frame point cloud and the scaffold reference point cloud to match the point cloud position. For path planning, the required vector map was manually planned in the orchard point cloud to specify the path of the roadway, and finally, navigation was achieved through pure path tracking. Field tests showed that the accuracy of the NDT SLAM method reached 5 cm in each rank with a coefficient of variation less than 2%. Additionally, the navigation system had a high positioning heading accuracy with a deviation within 1° and a standard deviation of less than 0.6° when moving along the path point cloud at a speed of 1.0 m/s in a Y-trellis pear orchard. The lateral positioning deviation was also controlled within 5 cm with a standard deviation of less than 2 cm. This navigation system has a high level of accuracy and can be customized to specific tasks, making it widely applicable in trellis orchards with autonomous navigation pesticide sprayers.

To address the issue of low-density canopy in greenhouse crops affecting the robustness and accuracy of simultaneous localization and mapping (SLAM) algorithms, Tan et al. proposed a map construction and localization method based on spatial down sampling with multiline LiDAR using the Cartographer framework. Focusing on suspended tomato plants grown in greenhouses as the research subject, an adaptive filtering point cloud projection (AF-PCP) SLAM algorithm was developed. The results demonstrated that compared to the Cartographer algorithm, the AF-PCP SLAM algorithm increased the average mapping area of crop rows by 155.7%. The mean error and coefficient of variation for crop row length were 0.019 m and 0.217%, respectively. Additionally, the average maximum void length was measured at 0.124m while the average relative localization errors at speeds of 0.2 m/s, 0.4 m/s, and 0.6 m/s were found to be 0.026 m, 0.029 m, and 0.046 m respectively; all with standard deviations less than or equal to 0.06 m. The proposed algorithm reduced average localization error by approximately79 .9% when compared to track deduction algorithms. Thus, it can be concluded that even in low-density canopy environments within greenhouses, the AF-PCP SLAM algorithm is capable of precisely mapping and localizing robots.

## Conclusion

The innovative advancement of chemical application technology for horticultural crops has resulted in reduced pesticide usage, improved operational efficiency, cost reduction, and risk mitigation associated with plant protection operations. We believe that this Research Topic will be a valuable resource for readers seeking to understand the latest advancements in chemical application technology for horticultural crops and their practical implications on precise spraying.

## Author contributions

XL: Conceptualization, Data curation, Investigation, Writing – original draft, Writing – review & editing. XLL: Funding acquisition, Project administration, Writing – review & editing. AH: Writing – review & editing. JS: Validation, Writing – review & editing. TX: Validation, Writing – review & editing. YQ: Validation, Writing – review & editing.
